# Adolescent borderline personality traits and dyadic behavior shape mother-adolescent cortisol synchrony

**DOI:** 10.1186/s40479-023-00218-z

**Published:** 2023-04-12

**Authors:** Leonie Fleck, Anna Fuchs, Stefan Lerch, Eva Möhler, Julian Koenig, Franz Resch, Michael Kaess

**Affiliations:** 1 Department of Child and Adolescent Psychiatry, Centre for Psychosocial Medicine, University of Heidelberg, Heidelberg, Germany; 2 Institute of Psychology, University of Heidelberg, Heidelberg, Germany; 3 University Hospital of Child and Adolescent Psychiatry and Psychotherapy, University of Bern, Bolligenstrasse 111, 3000 Bern 60, Switzerland; 4 Department of Child and Adolescent Psychiatry, Saarland University Medical Center, Homburg, Germany; 5 Department of Child and Adolescent Psychiatry, Psychosomatics and Psychotherapy, Faculty of Medicine and University Hospital Cologne, University of Cologne, Cologne, Germany

**Keywords:** Cortisol, Synchrony, BPD, Adolescence, Mother–child interaction

## Abstract

**Background:**

Associations between parent and child cortisol levels (“cortisol synchrony”) are often reported and positive synchrony may mark dyadic regulation on a physiological level. Although dyadic behavior during interaction and adolescent borderline personality disorder (BPD) traits are linked with individual and dyadic regulatory capacities, little is known about how both factors influence parent-adolescent cortisol synchrony. We hypothesized that cortisol synchrony would differ depending on behavioral synchrony, i.e., smooth reciprocal dyadic interaction patterns, adolescent BPD traits, and their interactions.

**Methods:**

Multilevel state-trait modeling was implemented to investigate associations between concurrent mother-adolescent state cortisol and mother-adolescent average cortisol levels in a community sample of 76 mother-adolescent dyads. Three saliva samples were collected across interaction paradigms. Behavioral synchrony was observed, and adolescent BPD traits were evaluated using clinical interviews.

**Results:**

First, behavioral synchrony and absence of BPD traits were linked with positive associations between adolescent and maternal state cortisol (positive synchrony), BPD traits with negative associations (negative synchrony). When interaction effects were examined, results were more nuanced. In low-risk dyads (higher behavioral synchrony, no BPD traits) asynchrony was found. When risk (BPD traits) and resource (higher behavioral synchrony) were combined, synchrony was positive. Lastly, in high-risk dyads (lower behavioral synchrony, adolescent BPD traits), negative synchrony was observed. Average adolescent and maternal cortisol levels were consistently positively associated in dyads with higher risk.

**Conclusions:**

Positive dyadic interaction patterns are associated with positive state cortisol synchrony in mother-adolescent dyads and could buffer the effect of BPD traits, possibly supporting the process of physiological regulation.

**Supplementary Information:**

The online version contains supplementary material available at 10.1186/s40479-023-00218-z.

## Introduction

The hypothalamic–pituitary–adrenal (HPA) axis is an endogenous stress system under social regulation [1]. Through a cascade of endocrine activity and the release of cortisol, the HPA axis supports the body in case of environmental demands and threats [2]. Activation of the HPA axis is reliably provoked by social stressors such as negative judgement or conflict, and social support and high quality parenting reduce HPA dysregulation [1, 3, 4]. Social processes have the potential to elicit arousal and dysregulation in individuals and to support individuals in managing those states [5]. Individual physiological states and behaviors are co-constructed and synchronized in dyadic interactions, indicating that physiology and behavior in one affect physiology and behavior in the other [6]. Studies on behavioral regulatory processes in parents and children have emphasized their role in the development of children’s self-regulation [7]. Negative, maladaptive dyadic behavior is linked with child and adolescent mental disorder and poorer treatment outcomes [8, 9]. Recently, dyadic physiological regulation has gained attention, with scholars acknowledging that physiological regulation, too, needs to be conceptualized not as inherently individual but social [10, 11].

A disorder which is profoundly linked with deficits in interpersonal functioning and self-regulation [12, 13], severely impaired parent–child interactions [14, 15] and HPA axis functioning [16] is adolescent Borderline Personality Disorder (BPD). BPD specifically has been characterized as a “disorder of impaired social regulation” across the life span [17]. Thus, while parent–child regulatory processes in physiology and dyadic behavior are of central interest in research on child and adolescent mental disorders in general, these processes may be particularly relevant and potentially altered in adolescents with BPD traits and their parents. However, studies with this very focus are lacking.

### Dyadic conceptualization of regulatory processes: parent–child cortisol synchrony

A significant number of studies reports associations between parent and child cortisol levels [3, 4, 18, 19]. However, existing studies present a range of terms and analytic methods, making it difficult to draw conclusions across multiple studies [20]. To reduce variability and promote a common language in the field, we chose the frequently used term “cortisol synchrony” for dyadic cortisol associations. We define parent–child cortisol synchrony as a dynamic, within-dyad coordination of cortisol levels across time that is directly tied to an interpersonal process [20]. Thus, cortisol synchrony will be used to describe within-dyad, concurrent associations between mother and adolescent state cortisol. Significant synchrony therefore indicates that when a mother’s cortisol response is higher/lower at one time point, adolescent’s cortisol response is also higher/lower (at the same timepoint). At the same time, it is of interest whether these state associations are separate from overall dyadic similarity in cortisol levels [4]. In order to be able to examine both dynamic cortisol synchrony as well as associations between overall cortisol functioning, we implemented multilevel (MLM) mixed-effects modeling, also called state-trait modeling [21]. MLM state-trait modeling allows for parsing within-dyad (WD) effects of concurrent and dynamic cortisol state associations and between-dyad (BD) effects of overall/average cortisol associations [20, 22, 23]. Thus, WD and BD effects as well as their links with risk and resource factors can be examined in more detail [4, 22, 24]. In addition, we follow a ‘statistical’ approach detailing synchrony according to the direction of the observed associations, not attributing any valence to these types of synchrony per se [20]. Thus, positive synchrony indicates that when a mother’s cortisol is higher at one time point, adolescent’s cortisol is also higher (at the same timepoint), and vice versa, without indicating whether this type of synchrony is adaptive or maladaptive. Cortisol synchrony can further be negative, or not significant, suggesting asynchrony. Unfortunately, yet, little is known about different types of synchrony in different contexts or samples to be able to reliably determine which type of synchrony is adaptive or maladaptive, and more research is needed highlighting synchrony in diverse contexts and linking it with outcome measures [20, 22]. However, when synchrony is adaptive, it is suggested to serve the conservation of resources, and to support affiliative bonds and social processing, while maladaptive synchrony is thought to contribute to depletion of resources, to disrupt social affiliation, and increase the risk for psychopathology [10, 11, 25].

### Parent-adolescent cortisol synchrony and dyadic behavior

While the potential of the parent–child relationship in shaping stress and emotion regulation in early and middle childhood is widely recognized, less is known about adolescents and their interactions with parents [26]. During adolescence, a significant shift in social behavior emerges, where children strongly orient towards peers and the social group they belong to, and parent–child relationships change in content (e.g. more conflicts and negative affect [27]) and context (e.g. less time spent together [28]). Considering these changes, the lack of work examining parent–child synchrony in adolescence is a significant gap in literature [20]. Whereas prior studies suggest that positive cortisol synchrony supports processes like parent–child bonding, teaching and regulation in early and middle childhood, this may not be the case in adolescence [10, 20, 29]. As autonomy development is an important developmental task during adolescence, tight physiological coordination with parents may not be adaptive [30]. The few studies which have examined parent-adolescent cortisol in community [31–33] and low-income samples [26] reported positive associations in diurnal variations [31, 32] and over the course of laboratory visits [26, 33], suggesting that indeed there is cortisol synchrony beyond middle childhood.

Physiological and behavioral synchrony describe different levels of regulatory processes in a dyad. For a comprehensive understanding of dyadic regulation it is vital to examine both conjointly [20, 34]. Among the few studies integrating both physiology and behavior in their assessment of adolescents and their parents, [32] found that higher negative affect in either child or mother was associated with higher positive associations in parent-adolescent diurnal cortisol slopes [32]. Borelli et al. [25] investigated the influence of maternal overcontrol and context on parent–child synchrony in 9–12-year-old children and reported that when one adverse factor was present, positive synchrony was observed (i.e. at pretask only in dyads with higher maternal overcontrol, or following a stress task only in dyads lower in maternal overcontrol). For the recovery period, higher overcontrol was linked with negative synchrony, and the authors argued that they may have not captured a true recovery, but processing of the stress condition instead. These rather complex findings are mirrored in younger age groups, where a higher number of studies have yet produced inconsistent results. For example, positive cortisol synchrony has been found in sensitive mothers and their children [29, 35], whereas negative cortisol synchrony has been observed in less sensitive mothers and their children [35] and in mothers and their disorganized toddlers [19]. At the same time, positive cortisol synchrony has also been found in dyads with lower behavioral synchrony [36] and in dyads with higher maternal punitive parenting [37]. Thus, whether or when certain forms of cortisol synchrony go hand in hand with behavioral synchrony is still unclear [20]. More research drawing on clearly defined constructs, appropriate methodology and different age groups is needed to determine whether cortisol synchrony may differ depending on observed behavior.

### Parent-adolescent cortisol synchrony moderated by adolescent borderline personality symptoms

Adolescence is not only a particularly interesting developmental phase for the study of parent–child regulatory processes, but also a turning point for developmental psychopathology that is marked by heightened risk [38]. It thus represents an important period for the study of both social processes and mental disorder. BPD features can be reliably diagnosed in adolescence and implicate severely impaired interpersonal functioning and self-regulation [12, 13]. Based on Social Baseline Theory and the assumption that regulation is an interpersonal process [39], BPD has recently been described as the disorder of impaired social regulation [17]. Adolescents with BPD present with a pervasive pattern of instable relationships, self-image and affect [40], and BPD traits such as anger outbursts or impulsivity are primarily expressed in interpersonal contexts [41]. BPD seems further to be associated with dysregulated HPA axis functioning in adulthood, specifically, with elevated baseline levels and blunted reactivity to social stressors [16]. Thus, higher levels of adolescent BPD traits could influence parent-adolescent cortisol synchrony via a multitude of pathways such as dysregulated HPA-functioning and impaired dyadic behavior in the parent-adolescent dyad. Given the profound connection between BPD traits and regulatory impairment in interpersonal contexts [16, 17, 41], BPD traits may alter parent-adolescent cortisol synchrony even above and beyond the presence of other mental disorders, preventing positive effects of adaptive dyadic regulation or enhancing negative effects of dysregulation. However, despite a potential influence of adolescent BPD traits on cortisol synchrony in adolescence studies are lacking. To the best of our knowledge, there is only one study which has highlighted a role of child mental disorder in cortisol synchrony, focusing on preschool children with autism spectrum disorder [42]. Thus, there is much to learn about the nature of cortisol synchrony in adolescence and how it is shaped by adolescent mental disorder and borderline personality traits specifically.

### Present study

Our study had several aims. First, our goal was to add to the literature by examining cortisol synchrony in mother-adolescent dyads. Following the call to ensure a fit between definition of synchrony and analytical approach [23, 43, 44], we implemented MLM state-trait modeling [21] to determine presence and type of cortisol synchrony, a method matching our definition of synchrony as a WD process over the course of a dyadic interaction [20]. Furthermore, MLM state-trait modeling allows for parsing WD and BD effects [20, 23], allowing us to investigate both concurrent, dynamic parent-adolescent cortisol synchrony and average cortisol associations across the interaction, which may represent different dyadic processes [4]. Based on prior findings we expected to find *positive cortisol synchrony* in our sample [32, 33], our analyses regarding average cortisol associations were exploratory. Our second goal was to examine whether dyadic behavior would moderate parent-adolescent synchrony. As synchrony is defined as an interpersonal process and in line with prior studies using conversational paradigms in older children, we measured cortisol synchrony during actual social interaction [20, 26, 32]. We observed systemic features of the adolescent-parent-relationship, i.e. behavioral synchrony, which characterizes the dyadic atmosphere rather than the behavior of one or both partners separately. Behavioral synchrony as conceptualized in this study indexes reciprocal and mutually satisfying interactions and the absence of negative tension. In spite of overlap, this conceptualization can be differentiated from approaches which do not include emotional valence or adaptiveness of observed synchrony into their definition, such as movement synchrony or behavioral mimicry (see e.g. [45, 46]). As positive cortisol synchrony has been commonly found in prior work involving community samples [32, 33], and studies examining moderating effects of behavior on cortisol synchrony remain inconsistent [20], we hypothesized to find *positive cortisol synchrony* in dyads with higher levels of behavioral synchrony, and *asynchrony* or even *negative synchrony* in those with lower levels of behavioral synchrony. Our third goal was to examine whether adolescent BPD traits would moderate parent-adolescent synchrony. Since there is first evidence of positive synchrony in adolescent community samples [32, 33] and BPD has been described as a disorder of impaired social regulation specifically [17], we hypothesized altered (i.e. *negative or absent) cortisol synchrony* in adolescents with a higher number of BPD traits and their mothers. Fourth, we examined whether adolescent BPD traits and behavioral synchrony would interact to jointly moderate cortisol synchrony. We assumed higher levels of behavioral synchrony to buffer effects of BPD traits such that when BPD traits were present but behavioral synchrony was higher, *positive cortisol synchrony* would be observed. Lastly, as further analyses were beyond the scope of this manuscript, we included additional exploratory research questions in the supplement a) testing whether presence of adolescent mental disorder would moderate cortisol synchrony/average cortisol associations, b) whether adolescent BPD traits remained a significant moderator of cortisol synchrony when presence of other mental disorders was controlled for and c) whether mental disorder and behavioral synchrony would interact to moderate cortisol synchrony.

## Method

### Participants

We examined a community sample of 76 adolescent-mother dyads who participated in a longitudinal study on temperament and the development of BPD traits [47–49]. Adolescents (46% girls) were 14.0 (SD = 0) years old, mothers on average 48.2 years old (SD = 4.6). Seventy percent of mothers held a university degree, 20% had finished Intermediate Secondary School, and 10% held a University Entrance Diploma. Ninety-one percent of mothers reported to be in a relationship. Eighty-four percent of adolescents were in Grammar School, 15% in Intermediate Secondary School, and 1% visited other school types. Out of 101 mother–child pairs who were examined at five time points starting two weeks after birth, 76 families participated in the last assessment (T6) which included physiological and behavioral data collection. The 76 mother-adolescent dyads did not differ significantly from dyads who dropped out with respect to maternal education (*χ2*(2) = 2.27, *p* = 0.321) or maternal relationship status (*χ2*(2) = 3.54, *p* = 0.838), infant birth weight (*t*(99) = -0.74, *p* = 0.466) or infant sex (*χ2*(1) = 0.09, *p* = 0.767) at T1, however, mothers who continued to participate (*M* = 33.76 years at T1) where older than mothers who dropped out (*M* = 31.96 years at T1; *t*(99) = -2.14, *p* < 0.05).

### Procedure

Initially, mothers were recruited via local obstetric units and offices and newspapers. Inclusion criteria were full term delivery, infant weight > 2500 g, APGAR scores > 7 and good health of the baby during the first three postnatal doctoral exams. Exclusion criteria were inability to speak or understand the German language, acute maternal mental disorder, excessive smoking or alcohol consumption and the use of drugs or medication possibly risking fetal health. Mothers and adolescents were invited to a three-hour assessment, where clinical and socio-demographic interviews were conducted with adolescents while mothers filled in questionnaires in a separate room. Further, a parent–child interaction paradigm was administered, and cortisol samples were taken. Families were compensated 70 Euro for participation.

### Measures

#### Cortisol sample collection

Three saliva samples were taken from mothers and adolescents each at the same time over the course of the visit. At the beginning of the visit, mother and adolescent participated in a short interview assessing sociodemographic information, which was followed by clinical interviews (adolescents) and the completion of questionnaires (mothers). After approximately two hours, mothers and adolescents completed a five-minute resting baseline where they sat quietly in separate rooms, and immediately after the first cortisol sample (baseline) was taken. Two subsequent samples were collected ten minutes after mother-adolescent dyads had engaged in a ten-minute positive (positive interaction sample) and a ten-minute negative (conflict discussion sample) interaction, respectively. On average, the positive interaction sample was taken 22.57 min (SD = 0.57) and the conflict sample was taken 44.64 min (SD = 0.97) after the baseline sample. The average assessment time of day was 4:13 pm (SD 2:23 h). Salivette (Sarstedt, Germany) sampling devices were used for saliva collection. Families were instructed to refrain from drinking and eating for at least 60 min prior to the first sample. Saliva samples were stored uncentrifuged at − 20 °C until assayed at Dresden University of Technology. After thawing, salivettes were centrifuged at 3,000 rpm for 5 min, which resulted in a clear supernatant of low viscosity. Salivary concentrations were measured using commercially available chemiluminescence immunoassay with high sensitivity (IBL International, Hamburg, Germany). The intra and interassay coefficients for cortisol were below 9%, respectively. Average raw cortisol levels were 2.53 for mothers (SD = 1.62, min = 0.48, max = 11.25 nmol/l) and 2.53 for adolescents (SD = 1.66, min = 0.34, max = 10.06 nmol/l). Screening for outliers, nine cortisol samples were identified as outliers (> = 3SD from the respective time point mean). Out of these, two mothers showed outlier values on two sampling occasions, suggesting an abnormal cortisol pattern. The first mothers’ cortisol levels with a maximum of 8.14 nmol/l at baseline still fell into the suggested reference range for the respective time of day and maternal age [50] and there was no indication of faulty data. The second mothers’ cortisol levels with a maximum of 11.25 nmol/l at baseline were higher than the suggested reference range. However, excluding this case did not change the results and this dyad was thus kept in the analytic sample. Furthermore, to resolve skewness of raw cortisol levels, a natural log transformation was applied, and log-transformed values were used in analyses.

#### Behavioral synchrony

Behavioral synchrony was observed and rated based on the Coding Interactive Behavior system (CIB, (Feldman R: Coding interactive behavior manual, unpublished, https://ruthfeldmanlab.com/coding-schemes-interventions/)). Two videotaped, ten-minute dyadic interactions were coded; a positive interaction paradigm, where mothers and adolescents discussed and planned fun activities they would like to engage in together, and a conflict interaction paradigm, where mothers and adolescents discussed conflicts between them. Two main raters were trained and certified by the author of the measure and two additional raters were trained by them. Twenty-four dyads were rated by at least two raters with an inter-rater agreement 88% and Cohen’s kappa = 0.78.

The CIB version for parent–child conversational paradigms covers 56 behavioral codes which receive ratings from 1 (low) to 5 (high). There are two dyadic behavior scales: Dyadic reciprocity (reciprocity, compatibility, and fluency, *α* = 0.88) and dyadic negativity (tension and constriction, *α* = 0.77). In order to compute a behavioral synchrony variable, dyadic negativity was subtracted from dyadic reciprocity. Behavioral synchrony during the fun day planning and the conflict discussion correlated highly (*r* = 0.82, *p* < 0.001), indicating that dyads remain their individual interactive style and “rhythm” in both contexts. In order to reflect the global interactive style and emotional climate of the dyad, behavioral synchrony was therefore averaged across both tasks. Thus, behavioral synchrony was higher when dyadic reciprocity was higher and dyadic negativity was lower during both interactions.

#### Borderline personality disorder traits

BPD traits according to DSM-IV criteria were assessed with the Childhood Interview for Borderline Personality Disorder (CI-BPD; [51]). For this study, the CI-BPD was translated into German language by a professional agency. The CI-BPD evaluates each of the nine DSM-criteria as either “absent” (0 points), “probably present” (1 point) or “definitely present” (2 points) during the past two years. Two psychologists were trained for reliability by the author of the measure. Further, 20 of the interviews where double coded. Inter-rater agreement per symptom ranged from 80 to 100%, with an overall agreement of 93%. A variable indicating the number of BPD criteria (traits) was calculated for each adolescent.

### Analytic plan

We implemented multilevel state-trait modeling (MLM), which accounts for the nested structure of dyadic data [52]. Furthermore, MLM allows for simultaneous estimation of between-dyad (BD) and within-dyad (WD) effects, which offers several advantages to address current issues within the field of synchrony research. First, state-trait MLM allows to operationalize synchrony as a WD association between maternal and adolescent cortisol which is in line with our definition of cortisol synchrony as a dynamic, interpersonal process [20]. Further, it allows us to focus not only on WD associations and BD associations in maternal and adolescent average cortisol levels, but also to highlight how these two processes may look differently [15]. Due to our focus on adolescent BPD traits we chose to model maternal cortisol predicting adolescent cortisol, a common approach used in prior work [23, 44]. On the BD level, maternal average (trait) cortisol was calculated by grand-mean centering [21]. Consequently, when mothers had an average cortisol value of zero, their average cortisol over the course of the visit was equivalent to the sample average. On the WD level, in-the-moment associations were addressed by capturing whether mother and adolescent state cortisol responses coordinated at any given timepoint across the visit [22, 53]. Maternal state cortisol was calculated by subtracting each mother’s average cortisol from her concurrent cortisol value at that specific timepoint. Maternal state cortisol levels thus represented each mother’s fluctuations around her own cortisol average and a state cortisol value of zero was equivalent to this mother’s average cortisol [21]. A positive state cortisol value represented an increase in cortisol with respect to a mother’s average cortisol, and a negative state value cortisol indexed a decrease with respect to average cortisol. State and average cortisol predictors were set to predict ‘total’ adolescent cortisol levels. This allowed for simultaneous estimation of WD and BD effects in one model based on the assumption that outcome (adolescent cortisol) variance is composed of both interindividual and intraindividual variance [21].

The unconditional means model revealed an Intraclass Correlation of 89.8% and random effects were found to be significant, confirming the appropriateness of MLM. First, we examined relationships between maternal and adolescent cortisol and sampling time. Cortisol levels significantly declined from baseline to positive interaction (Mothers: *β* = -0.13, 95% CI[-0.172; -0.096]; Adolescents: *β* = -0.21, 95% CI[-0.275; -0.146]), and from positive interaction to conflict discussion (Mothers: *β* = -0.14, 95% CI[-0.175; -0.100]; Adolescents: *β* = -0.19, 95% CI[-0.250; -0.121]). To preserve parsimony and to account for cortisol changes as a function of time we thus included a continuous variable indexing time passed since the baseline sample (“time since baseline”) in all models instead of a categorical timepoint variable [21]. Since the start time of the experiment varied per dyad, we further included the planned covariate “time of day” in every analytic model to account for the influence of diurnal HPA-axis activity [2, 21]. Further, to examine cortisol synchrony and associations between maternal and adolescent average cortisol levels, we included maternal average and state cortisol levels predicting adolescent cortisol. Depending on the model in question, we further added a continuous variable determining the number of BPD traits for each adolescent (“BPD traits”) and/or a continuous variable depicting the level of dyadic behavioral synchrony across both interactions (“behavioral synchrony”) (Supplement Tables S3 and S2) and respective interactions (Table 1). “Number of BPD traits” and “Time of Day” were centered so that values of zero represented the average sample level. 76 dyads and 228 observations were included in analyses. Random intercept and random slope models were estimated using the lme4 package in R. Effect sizes were calculated using Cohen’s *f*^*2*^ (small 0.02, medium, 0.15, large 0.35) in Stata.

**Table 1:** Behavioral synchrony modulates the effects of adolescent borderline personality traits on cortisol synchrony

**Parameter**	**Estimate (SE)**	**95% CI**	**Cohen’s *f***^***2***^
***Fixed effects***			
(Intercept)	**0.92 (0.06)**	**[0.81, 1.03]**	
Time since Baseline^a^	**-0.01 (0.001)**	**[-0.01, -0.006]**	**0.464**
Time of Day^b^	**-0.12 (0.03)**	**[-0.18, -0.07]**	**0.001**
Behavioral Synchrony^b^	0.01 (0.07)	[-0.13, 0.15]	0.000
BPD traits^b^	**-0.10 (0.05)**	**[-0.20, -0.003]**	**0.004**
State CT^a^	0.19 (0.13)	[-0.06, 0.44]	0.015
State CT^a^ × Behavioral Synchrony^b^	**0.28 (0.11)**	**[0.05, 0.51]**	**0.038**
State CT^a^ × BPD traits^b^	-0.09 (0.09)	[-0.26, 0.09]	0.005
State CT^a^ × BPD traits^b^ × Behavioral Synchrony^b^	**0.44 (0.09)**	**[0.25, 0.63]**	**0.148**
Average CT^b^	**0.25 (0.12)**	**[0.02, 0.49]**	**0.019**
Average CT^b^ × Behavioral Synchrony^b^	**-0.41 (0.14)**	**[-0.69, -0.14]**	**0.008**
Average CT^b^ × BPD traits^b^	**0.35 (0.11)**	**[0.12, 0.57]**	**0.014**
Average CT^b^ × BPD traits^b^ × Behavioral Synchrony^b^	**-0.51 (0.21)**	**[-0.93, -0.10]**	**0.009**
***Random effects***			
Intercept	**0.20 (0.45)**	**[0.38, 0.53]**	
State Cortisol Slope	**0.07 (0.26)**	**[0.06, 0.56]**	

*Note.* Model fit: χ^2^(12) = 294.27. 228 Observations. Marginal R^2^ = 0.486. Maternal cortisol predicting adolescent cortisol. State CT = Maternal cortisol reactivity, Average CT = Maternal average cortisol. BPD traits = Number of Borderline Personality Traits. Unstandardized estimates are presented. × = interaction term. ^a^Level 1 predictor, ^b^Level 2 predictor. Significant parameters in bold

For the first assessment of the longitudinal study 14 years ago it was determined that, to detect medium effect sizes at α = 0.05 and power = 0.80, a sample size of *n* = 100 dyads should be targeted. With the current assessment we reached a high retention rate (*n* = 76 dyads). Sensitivity analysis was performed for the two main three-way interaction terms to identify the smallest effect the study was powered to find (See Supplement and Figure S1 for details). Results suggested there may not be enough power for resolving the three-way interaction effect on the between subject level (Average CT*BPD traits*Behavioral Synchrony). However, the study was powered to resolve the three-way cross level interaction effect (State CT*BPD traits*Behavioral Synchrony, see Supplement).

## Results

Log transformed average cortisol levels were *M* = 0.77 (*SD* = 0.54) for mothers and *M* = 0.72 (*SD* = 0.62) for adolescents, and state cortisol levels were *M* = 0.00 (*SD* = 0.15) for mothers and *M* = 0.00 (*SD* = 0.23) for adolescents. Mean behavioral synchrony behavior was *M* = 0.78 (*SD* = 0.78), with a minimum value of -1.52 and a maximum value of 1.96. For 16% (*n* = 12) of the adolescents, interviewers endorsed one BPD trait. Seven percent of adolescents (*n* = 5) had two traits, and again seven percent had three or more traits (*n* = 5). Thus, 71% (*n* = 54) had no BPD traits, and 29% (*n* = 22) had at least one BPD trait.

Correlations of study variables are presented in Supplement Table S1. There were no associations between maternal/adolescent cortisol levels for any demographic variable except maternal age, which was significantly associated with maternal baseline cortisol levels (*r* = 0.25, *p* < 0.05). No significant links were found for education, relationship status, child sex, smoking, alcohol consumption, hormonal contraception, days since first day of last period for female participants, recent sickness, physical activity or body mass index. None of the women were pregnant. There was intraindividual stability in cortisol: Maternal baseline cortisol was significantly associated with cortisol after the positive interaction (*r* = 0.93, *p* < 0.01) and after the conflict discussion (*r* = 0.92,* p* < 0.01). Adolescent baseline cortisol was significantly associated with cortisol after the positive interaction (*r* = 0.92, *p* < 0.01) and after the conflict discussion (*r* = 0.83,* p* < 0.01). Further, maternal and adolescent cortisol levels were significantly correlated at baseline (*r* = 0.39, *p* < 0.01), positive interaction (*r* = 0.36, *p* < 0.01), and conflict discussion (*r* = 0.35, *p* < 0.01).

### Multilevel state trait modeling

#### Baseline model: are maternal and adolescent cortisol levels associated?

The baseline model included time since baseline, time of day, maternal state and average cortisol as predictors. Adolescent cortisol was significantly predicted by time since baseline (*β* = -0.01, 95% CI [-0.098, -0.006]) and time of day (*β* = -0.13, 95% CI [-0.190, -0.074]). We did not find cortisol synchrony in the total sample of 76 mothers and adolescents, as maternal state cortisol did not significantly predict adolescent state cortisol (*β* = 0.16, 95% CI [-0.149, 0.472]). Further, maternal average cortisol did not predict adolescent cortisol. There was, however, considerable intercept and slope variability between dyads (random intercept *β* = 0.24, 95% CI [0.422, 0.587]; random slope *β* = 0.45, 95% CI [0.410, 0.946]). Thus, there were substantial between-person differences in the association between average cortisol for mothers and adolescents, and substantial between-person differences in the within-person association between maternal and adolescent state cortisol, a variability which could potentially be explained by moderators [4].

#### Are cortisol synchrony and average cortisol associations moderated by behavioral synchrony?

##### State cortisol

Behavioral synchrony significantly moderated cortisol synchrony (*β* = 0.31, 95% CI [0.02, 0.60], see Supplement Table S2) such that when behavioral synchrony was *higher* (> 0.69 centered behavioral synchrony), maternal state cortisol positively predicted adolescent state cortisol. More precisely, when mothers showed a decrease in cortisol with respect to average at any given timepoint, adolescents showed a decrease with respect to average at the same timepoint, and vice versa.

##### Average cortisol

Behavioral synchrony also moderated the effects of maternal average cortisol (*β* = -0.29, 95% CI [-0.58, -0.003], see Supplement Table S2). Maternal average cortisol positively predicted adolescent average cortisol only when behavioral synchrony was *lower* (< -0.21 centered behavioral synchrony). Thus, higher maternal cortisol across the interaction was linked with higher adolescent cortisol across the interaction, and lower maternal cortisol across the interaction was linked with lower adolescent cortisol for dyads lower in behavioral synchrony.

#### Are cortisol synchrony and average cortisol associations moderated by adolescent BPD traits?

##### State cortisol

Adolescent BPD traits significantly moderated cortisol synchrony (β = -0.35, 95% CI [-0.53, -0.17], see Supplement Table S3). Simple slope analysis revealed that maternal state cortisol significantly predicted adolescent state cortisol when adolescents either had no BPD traits (*β* = 0.34; p < 0.05) or at least three BPD traits (Three traits: *β* = -0.68; p < 0.05). Synchrony direction differed depending on BPD traits: When adolescents had no BPD traits, synchrony was *positive*. Thus, decreases in maternal cortisol with respect to average were linked with decreases in adolescent cortisol at any given timepoint and increases in maternal cortisol were linked with increases in adolescent cortisol. For adolescents with three or more BPD traits, synchrony was *negative.* When mothers increased/ decreased their cortisol levels at any given timepoint, adolescents showed the opposite pattern.

##### Average cortisol

There were no significant effects for maternal average cortisol predicting adolescent average cortisol (β = 0.21, 95% CI [-0.03, 0.45], see Supplement Table S3).

#### Does behavioral synchrony modulate the effects of adolescent BPD traits on cortisol synchrony and average cortisol associations?

##### BPD traits and state cortisol

**Fig. 1:**
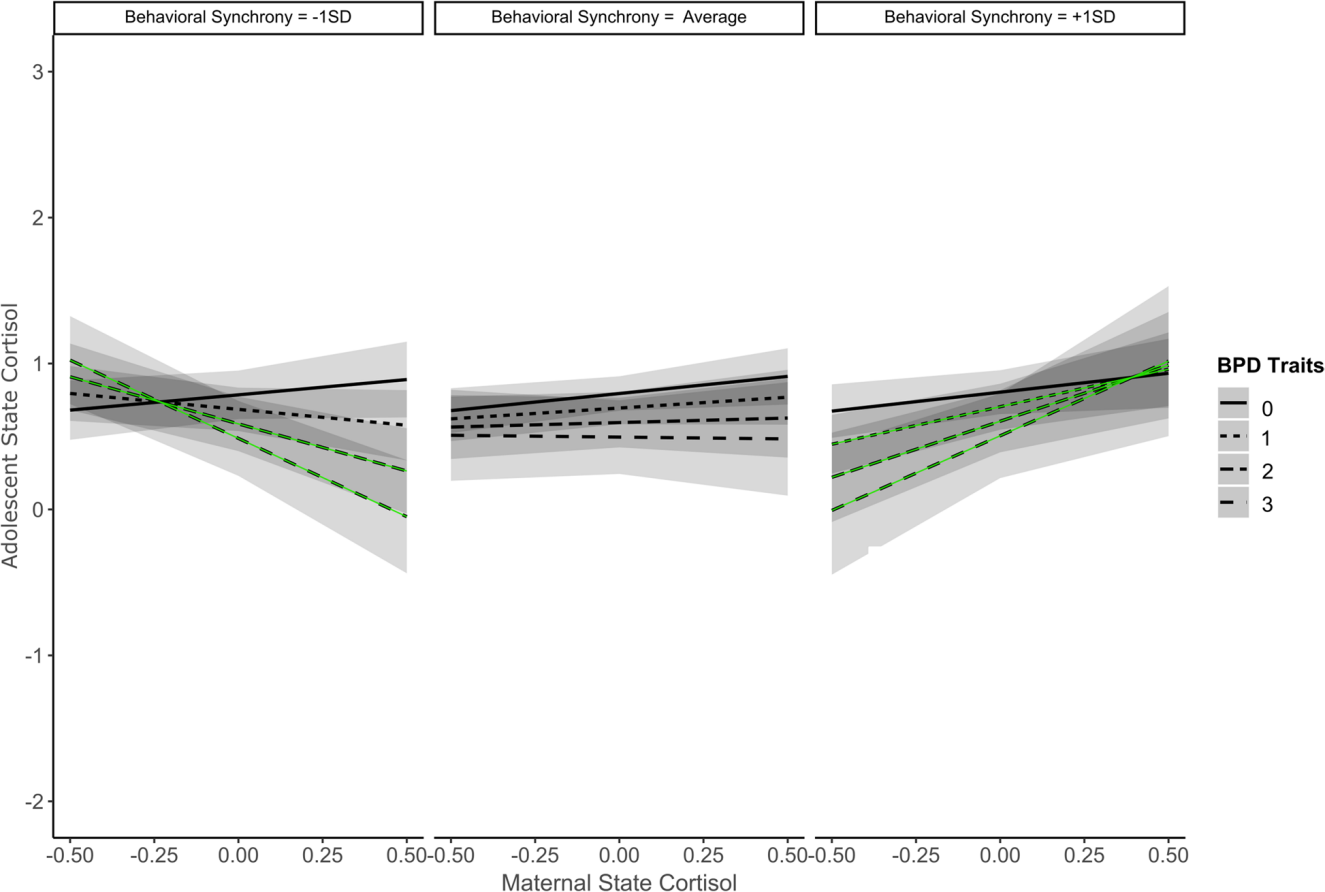
Adolescent Borderline Personality Traits and Behavioral Synchrony Moderate Cortisol Synchrony. *Note.* Green lines: *p* < .05; black lines: *p* > .05. ± 1SD = Above/below one standard deviation. Negative cortisol synchrony when behavioral synchrony was lower and adolescents had at least two BPD traits. Positive cortisol synchrony when behavioral synchrony was higher and adolescents had at least one BPD trait

Behavioral synchrony modulated the way BPD traits shaped cortisol synchrony (Table 1). Specifically, dyadic behavior made a difference when BPD traits were present: When behavioral synchrony behavior was *lower* (-1SD) and adolescents had at least two BPD traits, maternal state cortisol negatively predicted adolescent state cortisol (*negative synchrony*). However, when behavioral synchrony was *higher* (+ 1SD) and adolescents had at least one BPD trait, maternal state cortisol positively predicted adolescent state cortisol (*positive synchrony*). When adolescents had no BPD traits, and independent from the level of behavioral synchrony, asynchrony in cortisol was observed (Fig. 1). There was, however, trend-level positive synchrony in adolescents without BPD traits and their mothers when the dyad had average to higher behavioral synchrony (*p* < 0.10).

##### BPD traits and average cortisol

**Fig. 2:**
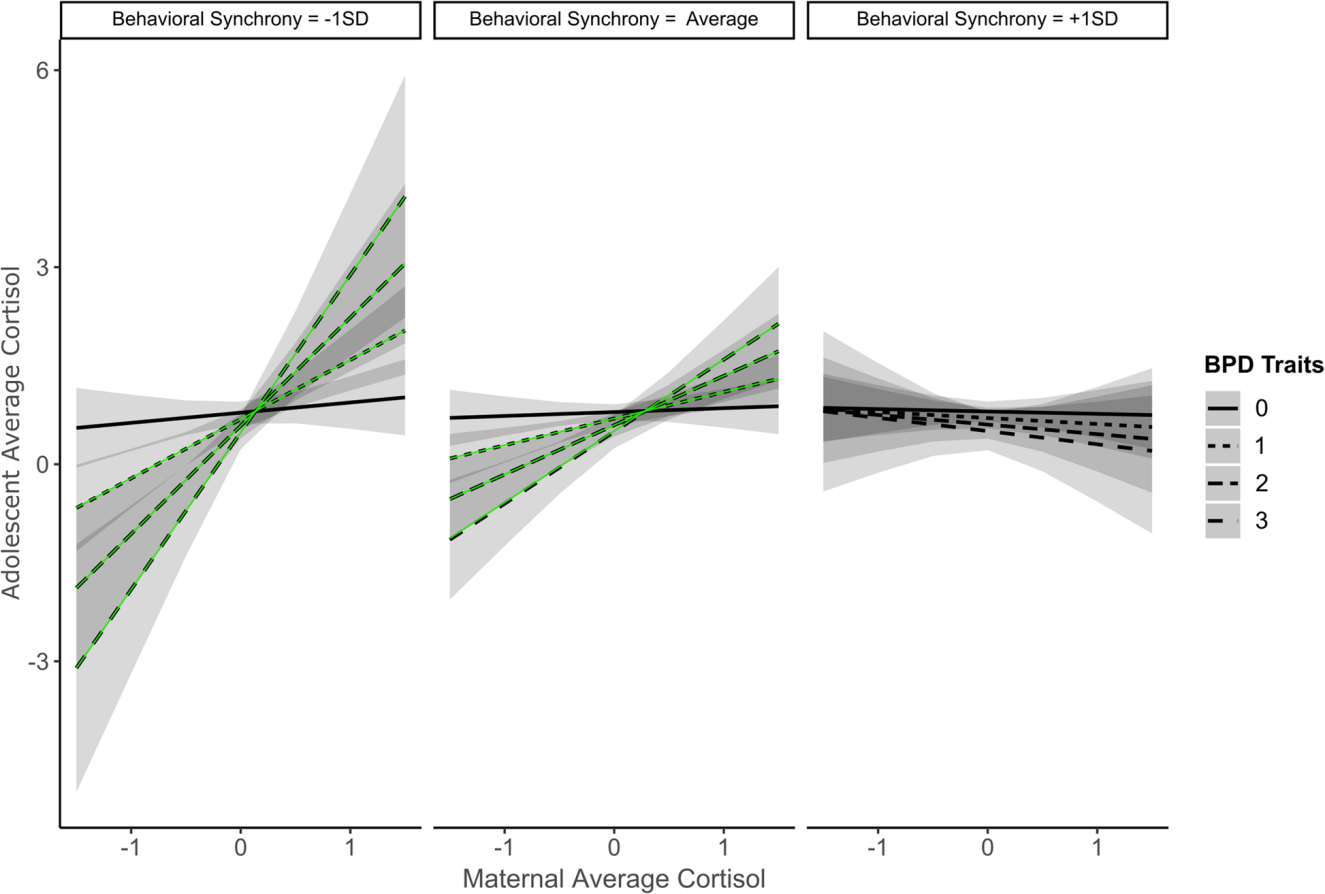
Adolescent Borderline Personality Traits and Behavioral Synchrony Moderate the Link between Maternal Average CT and Adolescent Average CT. *Note.* Green lines: *p* < .05; black lines: *p* > .05. ± 1SD = Above/below one standard deviation. Positive cortisol synchrony when behavioral synchrony was average or lower and adolescents had at least one BPD trait

Behavioral synchrony further shaped how maternal cortisol and BPD traits predicted adolescent average cortisol (Table 1; Fig. 2). When behavioral synchrony was higher (+ 1SD), maternal cortisol did not predict adolescent cortisol irrespective of adolescent BPD traits. When behavioral synchrony was lower (-1SD) or average level, however, results changed depending on BPD traits: For adolescents with at least one BPD trait, maternal average cortisol positively predicted adolescent average cortisol. There was no such effect when adolescents had no BPD traits. Maternal average cortisol positively predicted adolescent average cortisol when behavioral synchrony was lower. In addition, for adolescents with at least one BPD trait, maternal average cortisol significantly and positively predicted adolescent average cortisol. Lastly, main effects showed that the higher the number of BPD traits, the lower adolescent average cortisol, and the higher maternal average cortisol, the higher adolescent cortisol.

### Results summary

#### State cortisol

When behavioral synchrony and BPD traits were examined individually, both factors were meaningful moderators of mother-to-adolescent cortisol synchrony. *Positive* cortisol synchrony was found in dyads with higher behavioral synchrony and in dyads where adolescents did not have any BPD traits. *Asynchrony* was observed in dyads lower in behavioral synchrony and in dyads where adolescents reported one or two BPD traits. Importantly, when adolescents reported at least three BPD traits, *negative* synchrony was found.

When combined in one model, behavioral synchrony and BPD traits jointly shaped cortisol synchrony and results were more nuanced. When adolescents reported more than one BPD trait, but behavioral synchrony was higher, cortisol synchrony was *positive*. *Asynchrony* in cortisol was found regardless of BPD traits when behavioral synchrony was average. Finally, *negative* synchrony was observed when behavioral synchrony was lower and adolescents reported at least one BPD trait.

#### Average cortisol

Across all models, maternal average cortisol positively predicted adolescent average cortisol under conditions of risk only, i.e., lower behavioral synchrony and lower behavioral synchrony combined with at least one adolescent BPD trait. Thus, when behavioral synchrony was higher, across all models, average cortisol levels were not significantly linked. Furthermore, even if behavioral synchrony was lower, when adolescents did not report any BPD traits, maternal and adolescent average cortisol were not significantly associated.

## Discussion

Our study aimed to shed light on associations between concurrent, dynamic “state” and overall, “average” cortisol levels in adolescents and their mothers, which are theorized to indicate interpersonal physiological regulation. MLM state-trait modeling [21] allowed us to parse these WD and BD effects. Behavioral synchrony and BPD traits were relevant factors shaping cortisol synchrony, and our results mirror prior findings indicating that patterns of cortisol synchrony change depending on both risk and resources within a dyad [3, 4, 18, 19, 29, 42]. However, we observed differences in cortisol synchrony patterns depending on whether behavior and BPD traits were examined separately or conjointly and whether their interactions were probed.

When behavioral synchrony and BPD traits were examined separately, in line with our hypotheses, positive cortisol synchrony was linked with higher behavioral synchrony, whereas negative cortisol synchrony was linked with BPD traits in adolescents. These results are in line with studies suggesting that positive cortisol synchrony occurs in context of adaptive dyadic interaction, could be a marker of healthy parent–child co-regulatory processes and may support them on a physiological level [29, 35].

In line with our hypothesis, a higher number of BPD traits in adolescents was consistently linked with negative synchrony. BPD as a “disorder of social regulation” [17] is often characterized by severe difficulties in interpersonal relationships including the parent–child relationship, and behavioral and physiological regulatory impairments such as elevated baseline cortisol and blunted cortisol reactivity to social stressors [12, 13, 16]. Social impairments that are also observed in adolescent BPD are highly relevant factors for coregulatory processes in a dyad [22, 24, 42]. In addition, a recent study examining infant attachment as a moderator of mother–child cortisol synchrony found negative synchrony in dyads with disorganized children, with mothers showing decreases and infants showing increases in cortisol over time [19]. Insecure or disorganized attachment has been hypothesized to be a major predisposing factor for BPD pathology, and a recent study reported that young adults with BPD had a greater likelihood to exhibit disorganized interactions with their mothers than adults with other or no diagnoses [15]. Deficits in attachment quality may thus be one important factor behind the moderating effect of BPD traits on cortisol synchrony.

For adolescents with BPD traits, in our sample mainly characterized by impulsivity, self-harm/suicidality and uncontrollable anger [48], adaptive co-regulatory processes in behavior and physiology during social interaction could function as important stabilizers supporting adolescent self-regulatory systems. In our community sample, mothers and adolescents showed a constant decrease in cortisol on average. This decrease was especially pronounced in adolescents without BPD traits but was also observed in mothers and, albeit to a lesser extent, in adolescents with BPD traits. A decrease in maternal cortisol levels, on average, thus seemed to predict decreasing cortisol levels in adolescents without BPD traits and increasing cortisol levels in adolescents with BPD traits. This may suggest that adolescents with BPD traits could not adequately benefit from adaptive maternal physiological responses and might be an indicator of insufficient co-regulation on a physiological level. However, in order to determine whether negative synchrony in adolescents with BPD traits and their mothers is in fact maladaptive, further studies linking cortisol synchrony and adolescent outcome are indicated. In addition, it remains unclear whether these alterations in physiological synchrony are a consequence of adolescent BPD traits or may contribute to their development. Longitudinal studies on the pathogenesis of BPD traits in at-risk populations are needed to examine the role of co-regulatory processes over time.

Importantly however, while results based on separate models for behavioral synchrony and BPD indicated cortisol synchrony to be positive in context of higher behavioral synchrony, and absent or negative in context of lower behavioral synchrony and BPD traits, results were somewhat more nuanced when behavior and BPD traits were examined in one model and interaction effects were probed. In dyads with the lowest risk (higher behavioral synchrony and absence of BPD traits) we did not observe significant cortisol synchrony. In dyads where we observed higher positive synchrony as a resource, but adolescents reported at least one BPD trait, positive cortisol synchrony was found. Lastly, in dyads with two combined risk factors (lower behavioral synchrony and adolescent BPD traits), negative synchrony was observed. Interestingly, our finding of asynchrony in low-risk dyads (higher behavioral synchrony and absence of BPD traits) during potentially stressful situations could make a case for the notion that in adolescence, synchrony may not always be adaptive or developmentally normative [30]. Similarly, when the risk factor BPD traits was paired with the resource of higher behavioral synchrony, positive cortisol synchrony was found, suggesting that in these dyads, a decrease in maternal cortisol may indeed be linked with a decrease in adolescent cortisol. Hence, whereas it is theorized that individuals with BPD pathology do not adequately benefit from social regulation [17], adolescents with BPD traits were responsive to the effects of behavioral co-regulation with regard to HPA axis activity.

Cortisol synchrony seems to depend on dyadic behavior especially when adolescents report BPD traits. Higher levels of behavioral synchrony are characterized by mutual adaption and low tension. Behavioral synchrony may act as a dyadic buffer, allowing for adaptive co-regulatory processes and balancing out some of the regulatory and social impairments of BPD traits. In this case, positive cortisol synchrony may be an adaptive process. This could also inform interventions about possible mechanisms of successful co-regulation. It will be of interest to determine if this buffering effect is also observable in clinical samples, where the resource of behavioral synchrony in parent–child dyads might not always be present and has to buffer against higher levels of BPD pathology. Parent–child interventions could focus on establishing higher behavioral synchrony, and also movement synchrony in the therapist-patient dyad may be an indicator of therapeutic success [45]. At the same time, a consistent finding throughout all models showed negative cortisol synchrony in dyads where adolescent BPD traits were combined with lower levels of behavioral synchrony. In these cases, maladaptive dyadic behavior may even have exacerbated regulatory difficulties on a physiological level. Thus, negative cortisol synchrony may indicate dysfunctional dyadic regulatory processes. However, future longitudinal studies will have to examine whether negative cortisol synchrony is in fact associated with maladaptive child outcome such as emotion dysregulation, negative affect during interaction or more BPD traits.

Lastly, we were able to disentangle patterns of concurrent, dynamic parent-adolescent synchrony and average cortisol associations. While occurrence and form of state cortisol associations changed depending on which risk and resources where at play, associations between average cortisol in mothers and adolescents provided a consistent picture across all models. Maternal average cortisol was positively associated with adolescent average cortisol under conditions of higher risk (lower behavioral synchrony, adolescent BPD traits), and there was no significant association in dyads with higher positive dyadic behavior or when adolescents did not report any BPD traits. These consistent findings may suggest that mother-adolescent linkage in average cortisol represents an indicator of risk.

### Limitations

Despite its strengths, our study is not without limitations. Due to only three cortisol measurements, we were limited in our analytic approach and were unable to investigate nonlinear associations between mother and adolescent cortisol reactivity. Sensitivity analysis revealed that while the study was powered to find significant effects of state cortisol in three-way interactions, this was not the case for average cortisol. Thus, futures studies would benefit from a bigger sample size. Furthermore, despite all its advantages, concurrent state-trait MLM does not allow for examination of mother-to-adolescent or adolescent-to-mother directionality. Our study procedure was designed to elicit stress responses following a mother-adolescent conflict discussion. However, as reported in other studies (e.g. [26]), participants on average showed a steady decline in cortisol levels, suggesting participants habituated over time. While we were still able to examine mother-adolescent cortisol synchrony, our findings seem to suggest that conflict discussions may not be an adequate context to elicit cortisol responses to stress in parents and adolescents. Next, due to the community-based sample, BPD trait frequencies where rather low. Whereas even single BPD traits have been shown to be of clinical significance [54, 55], and their potential impact on physiological regulation is also supported by the current study, we cannot be sure that results generalize to clinical populations. Replications in higher-risk samples are therefore warranted. Further, as this was a longitudinal study focusing on mothers, we did not include fathers in our assessments. However, the one study focusing on parent–child cortisol synchrony in context of child disorder reported differences in cortisol synchrony in mother- and father-child dyads [42]. Moving forward, it will be important to investigate how synchrony differs in mother- vs. father-adolescent dyads [22, 23, 42].

## Conclusions

Our findings suggest that in the context of child regulatory difficulties that are reflected by the presence of different BPD traits, behavioral synchrony as a dyadic resource may act as a buffer in the context of physiological co-regulation. The interplay of risk and resources might explain seemingly inconsistent findings in the literature. Future studies should integrate both mental disorder, BPD traits specifically, and dyadic behavior as moderators of synchrony and replicate especially in higher risk samples how these factors shape cortisol synchrony distinctly and conjointly. Further, while we were able to show differences in cortisol synchrony depending on dyadic behavior and BPD traits, further research will have to elucidate how these differences indeed relate to child outcome. The question arises whether positive synchrony, which we observed in dyads characterized by higher behavioral synchrony and BPD traits, is adaptive in terms of successful co-regulation and adolescent healthy development. This could also inform interventions about potential mechanisms of successful co-regulation. Similarly, it will be important to highlight whether negative cortisol synchrony in context of adolescent BPD traits is maladaptive and whether attachment quality may be of relevance in this context. The examination of both behavioral and physiological synchrony in development and persistence of symptoms indicating impulsiveness and regulatory difficulties could be a fruitful future direction of research.

## Supplementary Information

**Additional file 1.**

## Data Availability

The dataset used during the current study is available from the corresponding author on reasonable request.

## Abbreviations

CIB: Coding Interactive Behavior
CI-BPD: Childhood Interview for Borderline Personality Disorder
CT: Cortisol
BD: Between-dyad
BPD: Borderline Personality Disorder
HPA-axis: Hypothalamic–pituitary–adrenal axis
MLM: Multilevel Modelling
WD: Within-dyad
