# Supplementary material for: Adolescent borderline personality traits and dyadic behavior shape mother-adolescent cortisol synchrony
Source: Borderline Personal Disord Emot Dysregul. 2023 Apr 12;10:12. doi: 10.1186/s40479-023-00218-z (PMC10091616; doi:10.1186/s40479-023-00218-z)
Supplement: Supplementary file 1 — Additional file 1. [file 40479_2023_218_MOESM1_ESM.docx]

**Supplement Material**

**Sensitivity Analysis**

This study had a given sample size, including 76 dyads with 3 observations per individuum. In order to calculate the smallest effect size the study was powered to find, we artificially tuned the effects of the two main three-way interactions (Average CT*BPD traits*Behavioral Synchrony, State CT*BPD traits*Behavioral Synchrony) and calculated power using bootstrapping. This allows interaction effects to be approximated by adding the estimated effects in the original sample to the artificially introduced effects. The effect sizes were thus not standardized but are reported in units of the three-way interaction terms. They are tuned in the direction of the observed effect, i.e. negative for the Average CT*BPD traits*Behavioral Synchrony interaction and positive for the State CT*BPD traits*Behavioral Synchrony interaction.

We used 1000 bootstrapped samples consisting of 76 dyads to approximate the power for each effect size. An additional contribution to the log-transformed child cortisol was added in each sample, proportional to the three-way interaction, and the model recalculated. This was done separately for both three-way interaction terms. Power corresponds to the proportion of significant three-way interaction effects within the 1000 analyses.

For a power of 0.8 at a significance level of α=.05, analyses revealed an effect size of about -0.7 for the Average CT*BPD traits*Behavioral Synchrony interaction and 0.3 for the State CT*BPD traits*Behavioral Synchrony interaction (Figure S1). This suggests there may not be enough power for resolving the three-way interaction effect of -0.5 on the between subject level (Average CT * BPD traits * Behavioral Synchrony). However, the study was powered resolve the three-way cross level interaction effect of 0.44 (State CT * BPD traits * Behavioral Synchrony interaction).

**Assessment of Other Mental Disorders**

The sixth version of the Mini International Neuropsychiatric Interview for Children and Adolescents (MINI-KID; Sheehan et al., 2010) was administered to adolescents. The MINI-KID is a structured diagnostic interview assessing mental disorders according to DSM IV in children and adolescents ages six to 17. It examines the 30 most common and clinically relevant disorders in pediatric mental health (Sheehan et al., 1998) and has been found to generate reliable and valid psychiatric diagnoses (Sheehan et al., 2010). For analysis, dichotomous factor “mental disorder” was created, indicating presence or absence of any mental disorder according to the MINI-KID. In total, 27.6% (n=21) of adolescents were evaluated to have a current mental disorder. Out of these, 19.7% percent of adolescents were diagnosed with any phobic or anxiety disorder, 9.2% with any behavioral disorder and 2.6% with any mood disorder.

**Additional Research Questions**

***Research Question 1:***

Are cortisol synchrony and average cortisol associations modulated by presence or absence of adolescent mental disorders?

**Results.** State cortisol: Presence or absence of adolescent mental disorder significantly moderated cortisol synchrony (Table S4) such that there was significant positive cortisol synchrony only in mothers and adolescents who did not have a disorder (*β*=.33; *p*<.05, Figure S2a). Average cortisol: In contrast, maternal average cortisol significantly predicted adolescent average cortisol only in adolescents with a mental disorder and their mothers (*β*=.37; p<.05; Table S4). In this group, higher maternal cortisol across all assessments was associated with higher adolescent cortisol across all assessments, and lower maternal cortisol was linked with lower adolescent cortisol across all assessments (Figure S2b). Further, there was a main effect of adolescent mental disorder such that if a disorder was present, adolescent cortisol was lower.

***Research Question 2:***

Do adolescent BPD traits remain a significant moderator of the association between adolescent and maternal cortisol above and beyond the influence of other relevant mental disorders?

**Results.** To compare the effects of BPD traits and other mental disorders on cortisol synchrony we included both main effects and their interactions with average and state cortisol in one model. The moderating effect of BPD traits (*β*=-.30; *p*<.01) and the main effect of mental disorder remained significant (*β*=-.31; *p*<.05). Again, we found positive cortisol synchrony in adolescents with no BPD traits and their mothers and negative cortisol synchrony in adolescents with at least three BPD traits and their mothers. Mental disorder remained negatively linked with adolescent average cortisol.

***Research Question 3***:

Does behavioral synchrony modulate the effects of presence or absence of adolescent mental disorders on cortisol synchrony and average cortisol associations?

**Results.** Mental Disorder and State Cortisol: Behavioral synchrony modulated the way presence or absence of mental disorder shaped cortisol synchrony (see Table S5, Figure S3). When behavioral synchrony was higher (+1SD) but adolescents had a disorder, *positive synchrony* was observed. When behavioral synchrony was higher and adolescents did not have a disorder, maternal and adolescent state cortisol were not significantly correlated (*asynchrony*). When behavioral synchrony was lower (-1SD) and adolescents had a disorder, *negative synchrony* was observed, whereas when behavioral synchrony was lower and adolescents did not have a disorder, *positive synchrony* was found. Mental Disorder and Average Cortisol: Mirroring findings reported in the prior mental disorder model, maternal average cortisol positively predicted adolescent average cortisol only when adolescents had a disorder (Figure S4). However, when behavioral synchrony was higher and adolescents had a disorder, there was asynchrony. Further, a positive main effect of maternal state cortisol emerged, as well as a main effect of disorder, suggesting that adolescents with mental disorder, on average, had significantly lower levels of cortisol.

**Results Summary**

*State Cortisol*

Mental disorder was a meaningful moderator of mother-to-adolescent cortisol synchrony. When mental disorder was examined without including behavior in the model, positive cortisol synchrony was observed in mothers and adolescents who did not have a disorder. However, after including behavioral synchrony as an additional moderator, positive cortisol synchrony was observed in dyads who showed higher behavioral synchrony and whose adolescents had a disorder. Asynchrony was found in the low-risk group of dyads with higher behavioral synchrony and adolescents who did not have a disorder, while negative cortisol synchrony was observed in the high-risk group of dyads with lower behavioral synchrony and adolescents who had a disorder.

Interestingly, any moderating effect of mental disorder did not remain significant when BPD traits were added to the model while the significant effect of BPD traits remained.

Negative cortisol synchrony in adolescents with at least three BPD traits and their mothers remained significant when presence of other mental disorders was controlled for.

*Average Cortisol*

Maternal average cortisol predicted adolescent average cortisol consistently and positively when adolescents had a mental disorder. However, when including behavioral synchrony as a moderator, this association remained significant only when dyads also showed lower behavioral synchrony. Thus, maternal and adolescent average cortisol were not significantly linked when either adolescents did not have a disorder, or adolescent had a disorder but behavioral synchrony was higher in the dyad.

**Tables**

**Table S1**

*Correlations Between Study Variables.*

| Variable | 1) | 2) | 3) | 4) | 5) | 6) | 7) | 8) | 9) | 10) |
| --- | --- | --- | --- | --- | --- | --- | --- | --- | --- | --- |
| 1) Average CT mother^1^ |  |  |  |  |  |  |  |  |  |  |
| 2) Average CT adolescent^1^ | 0.37** |  |  |  |  |  |  |  |  |  |
| 3) State CT mother^1^ | -0.01 | 0.00 |  |  |  |  |  |  |  |  |
| 4) State CT adolescent^1^ | 0.01 | 0.00 | 0.59** |  |  |  |  |  |  |  |
| 5) Number of BPD traits | -0.19 | -0.43** | -0.01 | -0.01 |  |  |  |  |  |  |
| 6) Mental Disorder (present/absent)^2^ | -0.13 | -0.37** | -0.01 | 0.00 | 0.52** |  |  |  |  |  |
| 7) Behavioral Synchrony | -0.04 | 0.04 | 0.00 | 0.00 | -0.10 | -0.20 |  |  |  |  |
| 8) Time of Day | -0.56** | -0.57** | 0.01 | 0.00 | 0.40** | 0.30** | 0.07 |  |  |  |
| 9) Time since Baseline | -0.02 | -0.05 | -0.74** | -0.68** | 0.04 | 0.03 | -0.01 | 0.02 |  |  |
| 10) Age Mother | 0.19 | 0.07 | 0.00 | 0.01 | -0.08 | -0.20 | 0.13 | -0.03 | 0.01 |  |
| 11) Sex Child (male) | -0.06 | 0.17 | 0.00 | 0.00 | 0.01 | -0.08 | -0.09 | -0.15 | 0.00 | 0.03 |

*Note.* CT=cortisol; ^1^logarithmized cortisol values; ^2^point-biserial correlation. **p*<.05, ***p*<.01.

**Table S2**

*Cortisol Synchrony Moderated by Behavioral Synchrony.*

| Parameter | Estimate (SE) | 95% CI | Cohen’s *f^2^* |
| --- | --- | --- | --- |
| *Fixed effects* |  |  |  |
| (Intercept) | **0.90 (0.06)** | **[0.78, 1.02]** |  |
| Time since Baseline ^1^ | **-0.01 (0.001)** | **[-0.01, -0.01]** | **0.515** |
| Time of Day ^2^ | **-0.13 (0.03)** | **[-0.18, -0.07]** | **-0.002** |
| Behavioral Synchrony ^2^ | 0.05 (0.07) | [-0.09, 0.20] | 0.000 |
| State CT ^1^ | 0.13 (0.15) | [-0.17, 0.44] | 0.006 |
| State CT ^1^ * Behavioral Synchrony ^2^ | **0.31 (0.15)** | **[0.02, 0.60]** | **0.023** |
| Average CT ^2^ | 0.20 (0.12) | [-0.05, 0.45] | 0.004 |
| Average CT ^2^ * Behavioral Synchrony ^2^ | **-0.29 (0.15)** | **[-0.58, -0.003]** | **-0.001** |
| *Random effects* |  |  |  |
| Intercept _µ0j_ | **0.23 (0.48)** | **[0.41, 0.57]** |  |
| State Cortisol Slope | **0.39 (0.63)** | **[0.36, 0.89]** |  |

*Note.* Model fit: χ^2^(7)=183.34, *p*<0.01. Maternal cortisol predicting adolescent cortisol. State CT=Maternal cortisol reactivity, Average CT=Maternal average cortisol. Unstandardized estimates are presented.  ^1^=Level 1 predictor, ^2^=Level 2 predictor. Significant parameters in bold.

**Table S3**

*Cortisol Synchrony Moderated by Adolescent Borderline Personality Traits*

| Parameter | Estimate (SE) | | 95% CI | Cohen’s *f^2^* |
| --- | --- | --- | --- | --- |
| *Fixed effects* |  |  | |  |
| (Intercept) | **0.91 (0.06)** | **[0.79, 1.03]** | |  |
| Time since Baseline ^1^ | **-0.01 (0.00)** | **[-0.01, -0.01]** | | **0.516** |
| Time of Day ^2^ | **-0.14 (0.03)** | **[-0.20, -0.08]** | | **0.007** |
| BPD Traits ^2^ | -0.08 (0.05) | [-0.18, 0.02] | | 0.001 |
| State CT ^1^ | 0.14 (0.15) | [-0.15, 0.42] | | 0.006 |
| State CT ^1^ * BPD traits ^2^ | **-0.35 (0.09)** | **[-0.53, -0.17]** | | **0.083** |
| Average CT ^2^ | 0.11 (0.13) | [-0.14, 0.37] | | 0.001 |
| Average CT ^2^ * BPD traits ^2^ | 0.21 (0.12) | [-0.03, 0.45] | | 0.001 |
| *Random effects* |  |  | |  |
| Intercept | **0.23 (0.48)** | **[0.41, 0.57]** | |  |
| State Cortisol Slope | **0.28 (0.53)** | **[0.24, 0.79]** | |  |

*Note.* Model fit: χ^2^(7)=207.44, *p*<0.01. Maternal cortisol predicting adolescent cortisol. State CT=Maternal cortisol reactivity, Average CT=Maternal average cortisol. Unstandardized estimates are presented. BPD traits=Number of Borderline Personality Traits. ^1^=Level 1 predictor, ^2^=Level 2 predictor. Significant parameters in bold.

**Table S4**

*Cortisol Synchrony Moderated by Other Mental Disorder*

| Parameter | Estimate (SE) | 95% CI | Cohen’s *f^2^* |
| --- | --- | --- | --- |
| *Fixed effects* |  |  |  |
| (Intercept) | **1.00 (0.07)** | **[0.87, 1.13]** |  |
| Time since Baseline ^1^ | **-0.01 (0.00)** | **[-0.01, -0.006]** | **0.517** |
| Time of Day ^2^ | **-0.13 (0.03)** | **[-0.19, -0.08]** | **0.006** |
| Mental Disorder (yes/no) ^2^ | **-0.32 (0.13)** | **[-0.56, -0.07]** | **0.003** |
| State CT ^1^ | **0.32 (0.16)** | **[0.01, 0.65]** | **0.026** |
| State CT ^1^ * Mental Disorder (yes/no) ^2^ | **-0.63 (0.26)** | **[-1.13, -0.11]** | **0.033** |
| Average CT ^2^ | -0.09 (0.16) | [-0.41, 0.20] | 0.000 |
| Average CT ^2^ * Mental Disorder (yes/no) ^2^ | **0.49 (0.21)** | **[0.08, 0.90]** | **0.004** |
| *Random effects* |  |  |  |
| Intercept | **0.21 (0.45)** | **[0.39, 0.54]** |  |
| State Cortisol Slope | **0.39 (0.62)** | **[0.34, 0.89]** |  |

*Note.* Model fit: χ^2^(7)=200.27, *p*<0.01. Maternal cortisol predicting adolescent cortisol. State CT=Maternal cortisol reactivity, Average CT=Maternal average cortisol. Unstandardized estimates are presented. ^1^=Level 1 predictor, ^2^=Level 2 predictor. Significant parameters in bold.

**Table S5**

*Behavioral Synchrony Modulates the Effects of Adolescent Mental Disorder on Cortisol Synchrony.*

| Parameter | Estimate (SE) | 95% CI | Cohen’s *f^2^* |
| --- | --- | --- | --- |
| *Fixed effects* |  |  |  |
| (Intercept) | **1.01 (0.07)** | **[0.88, 1.14]** |  |
| Time since Baseline ^1^ | **-0.01 (0.001)** | **[-0.01, -006]** | **0.530** |
| Time of Day ^2^ | **-0.12 (0.03)** | **[-0.18, -0.06]** | **0.000** |
| Behavior ^2^ | -0.01 (0.07) | [-0.15, 0.13] | 0.000 |
| Mental Disorder (yes/no) ^2^ | **-0.32 (0.13)** | **[-0.57, -0.07]** | **0.003** |
| State CT ^1^ | **0.31 (0.14)** | **[0.03, 0.59]** | **0.034** |
| State CT  ^1^ * Behavior ^2^ | -0.19 (0.14) | [-0.49, 0.10] | 0.012 |
| State CT ^1^ * Mental Disorder (yes/no) ^2^ | -0.38 (0.21) | [-0.80, 0,05] | 0.016 |
| State CT ^1^ * Mental Disorder (yes/no) ^2^ * Behavior ^2^ | **1.35 (0.26)** | **[0.83, 1.87]** | **0.176** |
| Average CT ^2^ | -0.05 (0.15) | [-0.36, 0.26] | 0.000 |
| Average CT ^2^ * Behavior ^2^ | -0.05 (0.18) | [-0.40, 0.31] | 0.000 |
| Average CT ^2^ * Mental Disorder (yes/no) ^2^ | **0.46 (0.20)** | **[0.06, 0.87]** | **0.003** |
| Average CT ^2^ * Mental Disorder (yes/no) ^2^ * Behavior ^2^ | -0.41 (0.28) | [-0.97, 0.15] | 0.001 |
| *Random effects* |  |  |  |
| Intercept | **0.19 (0.44)** | **[0.38, 0.52]** |  |
| State Cortisol Slope | **0.13 (0.35)** | [0.00, 0.63] |  |

*Note.* Model fit: χ^2^(12)= 281.36, *p*<0.01. Maternal cortisol predicting adolescent cortisol. State CT=Maternal cortisol reactivity, Average CT=Maternal average cortisol. Behavior=Behavioral Synchrony. Unstandardized estimates are presented. ^1^=Level 1 predictor, ^2^=Level 2 predictor. Significant parameters in bold.

**Figures**

**Figure S1.**

*Sensitivity Analysis for the two main three-way interaction terms.*


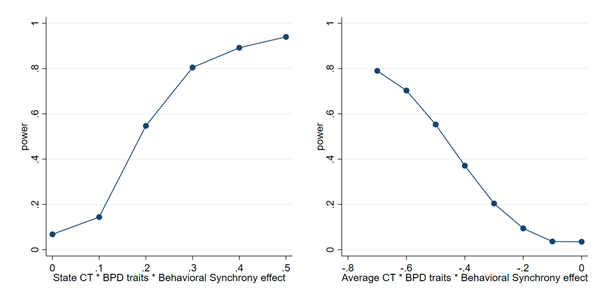


*Note.* Effect sizes of the three-way interactions were changed and power calculated via bootstrapping.

**Figure S2.**

*Presence or Absence of Adolescent Mental Disorders Moderates Cortisol Synchrony and Average Associations.*

*
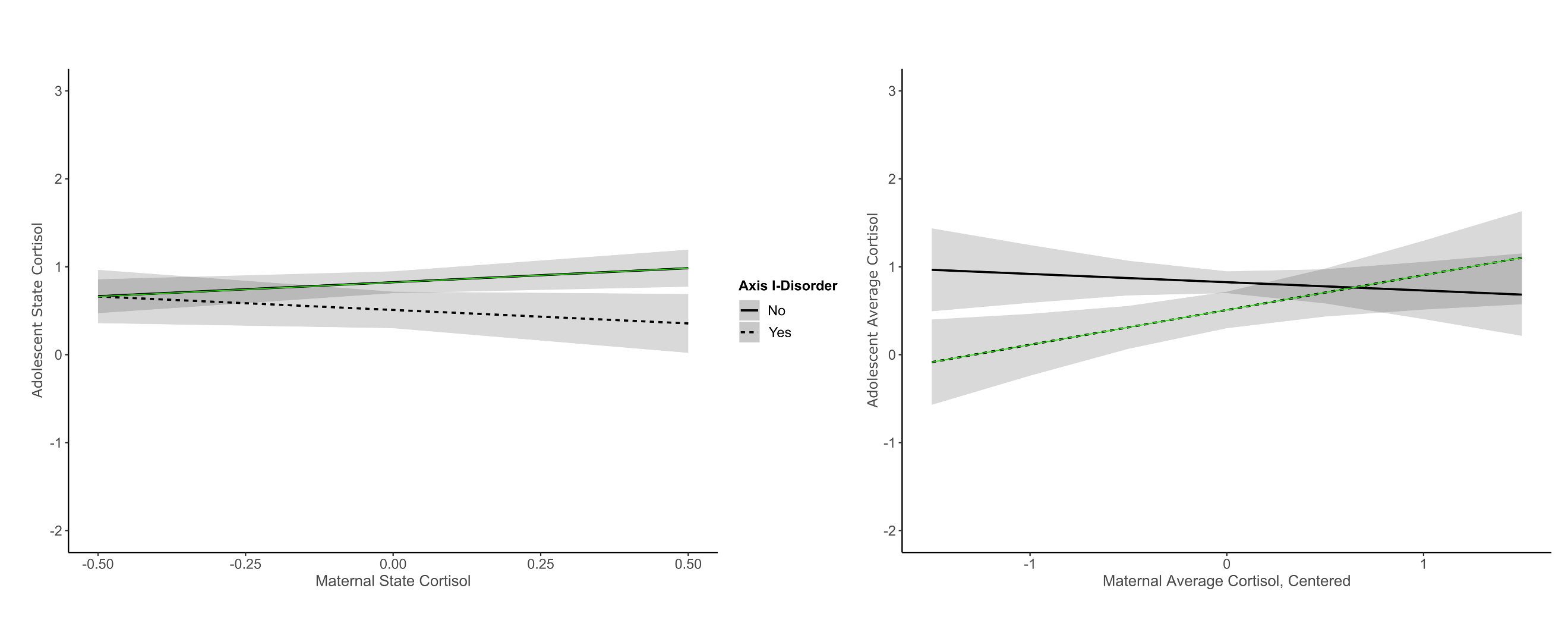
*

*Note.* Green line: *p*<.05; black line: *p*>.05. a) Significant when adolescents did not have a disorder b) Significant when adolescents had a disorder.

**Figure S3.**

*Adolescent Mental Disorders and Behavioral Synchrony Moderate Cortisol Synchrony.*

*
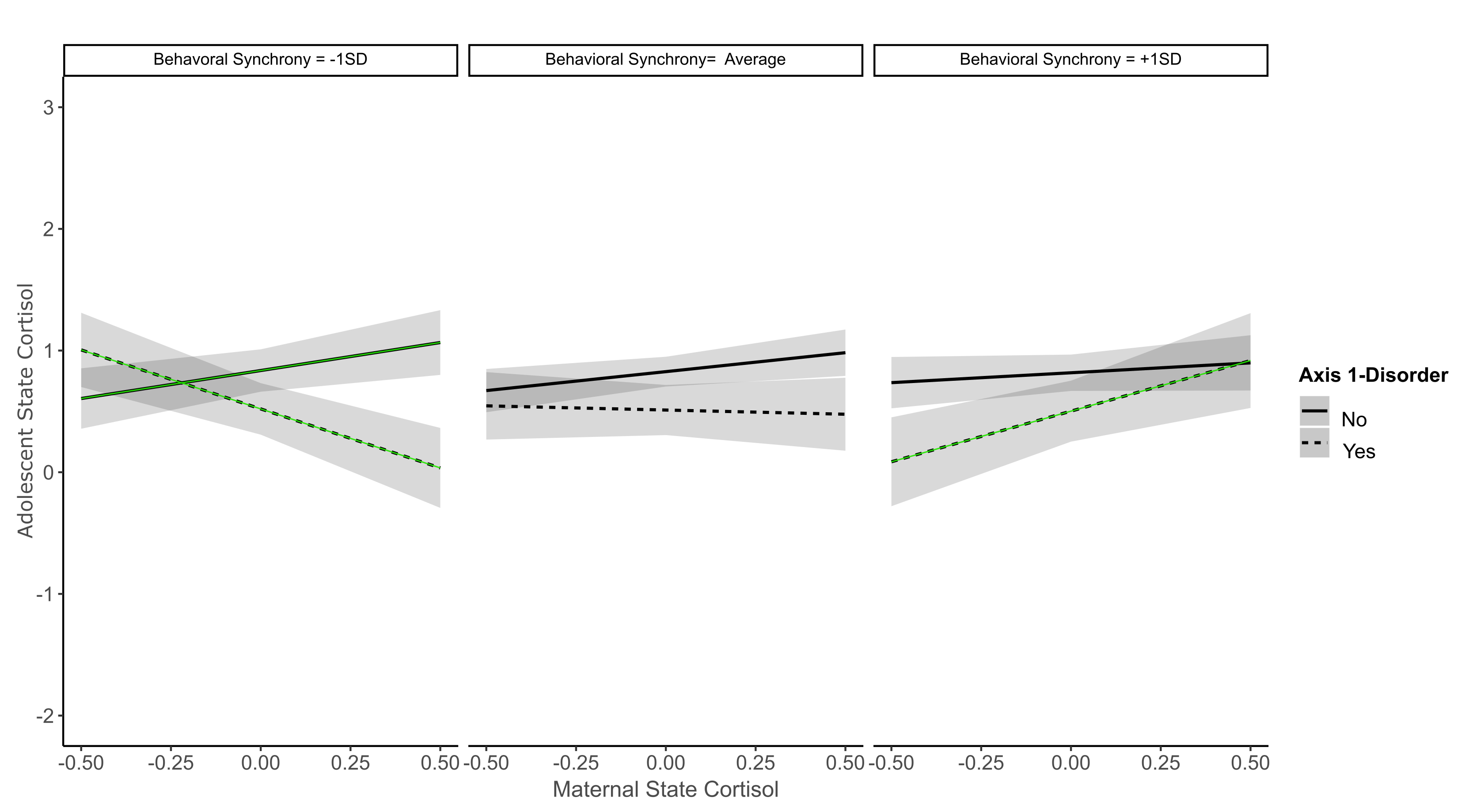
*

*Note.* Green lines: *p*<.05; black lines: *p*>.05. +/-1SD=Above/below one standard deviation. Negative cortisol synchrony when behavioral synchrony was lower, and adolescents had a disorder. Positive cortisol synchrony when a) behavioral synchrony was lower and adolescents did not have a disorder and b) when behavioral synchrony was higher, but adolescents had a disorder.

**Figure S4.**

*Presence or Absence of Adolescent Mental Disorder and Positive Dyadic Behavior Moderate the Link between Maternal Average CT and Adolescent Average CT.*

*
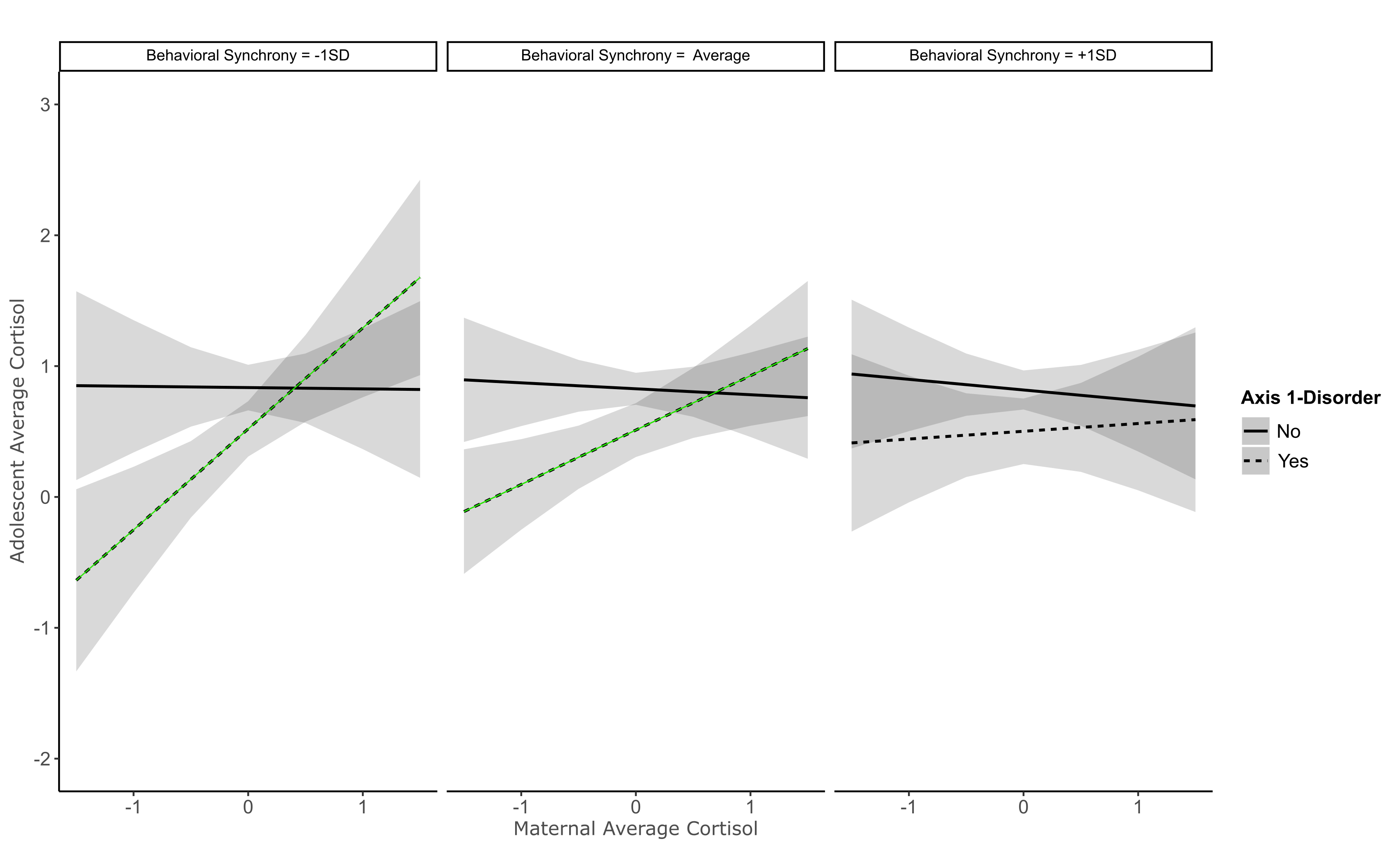
*

*Note.* Green lines: *p*<.05; black lines: *p*>.05. +/-1SD=Above/below one standard deviation. Positive average cortisol association when behavioral synchrony was average or lower and adolescents had a mental disorder.
